# Outbreak of Severe Respiratory Illness in an Assisted-Living Facility — Colorado, 2012

**Published:** 2013-03-29

**Authors:** Wendy Bamberg, Matthew Moore, Nimalie Stone, Joseph Perz, Raymund Dantes, Joyanna Wendt

**Affiliations:** Colorado Dept of Public Health and Environment; Div of Bacterial Diseases, National Center for Immunization and Respiratory Diseases; Div of Healthcare Quality Promotion, National Center for Emerging and Zoonotic Infectious Diseases; EIS officers, CDC

On May 28, 2012, the Colorado Department of Public Health and Environment (CDPHE) was notified of six cases of severe respiratory illness among 12 residents of an assisted-living facility (ALF) specializing in the care of elderly persons with dementia or memory loss. During May 22–27, 2012, five residents were hospitalized, and two developed invasive disease with *Streptococcus pneumoniae* (pneumococcal) bacteremia. *S. pneumoniae* is spread by airborne droplets and causes an estimated 175,000 hospitalizations and 50,000 cases of pneumococcal bacteremia each year. The case-fatality rate of pneumococcal bacteremia can be as high as 60% among the elderly.

CDPHE and CDC conducted an investigation to determine the extent of the outbreak and to assess the infection control capabilities at the facility. A probable case of pneumococcal disease was defined in a resident or staff member who received a diagnosis of pneumonia by a health-care provider during May 15–June 3, 2012. Confirmed cases met criteria for probable infection and also had *S. pneumoniae* isolated from a normally sterile site. CDPHE performed serotyping of culture isolates from confirmed cases.

Two confirmed and five probable cases of pneumococcal disease were identified; six patients (two with confirmed and four with probable pneumococcal disease) were residents, and one patient with probable pneumococcal disease was a staff member. Three of the six resident patients died. Median age of the seven patients was 80 years (range: 39–97 years) and all had received the 23-valent pneumococcal polysaccharide vaccine, consistent with guidelines from the Advisory Committee on Immunization Practices (1). The staff member had received pneumococcal polysaccharide vaccine because of a history of asthma.

All patients shared common areas in the ALF; the staff member’s responsibilities required close contact with all residents at the facility. Patients had symptom onset during May 19–27. The staff member had the earliest onset of respiratory symptoms and continued to work while symptomatic, raising concerns that the staff member might have introduced the infection into the facility. Although the ALF had an employee sick leave policy, staff members might not have been aware of the policy and its role in infection control. Additionally, the facility did not have a written infection control policy for maintaining minimum stocks of personal protective equipment such as gowns and face masks, and staff members were not aware such equipment should be worn to prevent person-to-person transmission of an unknown respiratory illness (2).

Isolates from both confirmed cases were identified as *S. pneumoniae* serotype 3 with indistinguishable antimicrobial resistance patterns. All residents were offered empiric postexposure chemoprophylaxis to reduce nasopharyngeal colonization with *S. pneumoniae*. All residents also were offered 13-valent pneumococcal conjugate vaccine (3). After careful consideration, public health officials did not identify additional benefits that could be gained from extending either prophylaxis or vaccination recommendations to the entire facility staff.

ALFs are community-based residential facilities that offer 24-hour supervision and also can provide supportive services such as medication management and dementia care (4). Considered the fastest-growing segment of long-term care, approximately 730,000 persons currently reside in the 31,000 licensed ALFs in the United States (5). Whereas hospitals and skilled-nursing facilities have federal regulatory standards for infection control and prevention programs (6), similar requirements currently do not exist for ALFs. Infection control requirements for ALFs vary among states, and ALF staff members might not have training in infection prevention and control. This outbreak in a vulnerable elderly population in a nonacute health-care setting highlights the importance of infection prevention training, guidance, and oversight for ALFs and their staffs.

To prevent future outbreaks of communicable illness in the Colorado ALF, CDC and CDPHE provided recommendations to increase support and awareness of existing sick-leave policies among staff members (e.g., not reporting to work when ill), and to develop and implement written infection control policies that include staff education, adequate availability and appropriate use of personal protective equipment, and recognition and reporting of disease outbreaks to public health authorities.
